# Research Recommendations for Improving Measurement of Treatment Effectiveness in Depression

**DOI:** 10.3389/fpsyg.2017.00356

**Published:** 2017-03-09

**Authors:** Kaloyan Kamenov, María Cabello, Mónica Nieto, Renaldo Bernard, Elisabeth Kohls, Christine Rummel-Kluge, José L. Ayuso-Mateos

**Affiliations:** ^1^Instituto de Salud Carlos III, Centro de Investigación Biomédica en Red, CIBERMadrid, Spain; ^2^Department of Psychiatry, Universidad Autónoma de MadridMadrid, Spain; ^3^Instituto de Investigación de La Princesa (IIS-IP), Hospital Universitario de La PrincesaMadrid, Spain; ^4^Department of Medical Informatics, Biometry and Epidemiology – IBE, Research Unit for Biopsychosocial Health, Ludwig-Maximilians-Universität MünchenMunich, Germany; ^5^Faculty of Medicine, Department of Psychiatry, Leipzig UniversityLeipzig, Germany

**Keywords:** depression, functioning, intervention, outcome measure, treatment effectiveness

## Abstract

**Background:** Despite the steadily escalating psychological and economic burden of depression, there is a lack of evidence for the effectiveness of available interventions on functioning areas beyond symptomatology. Therefore, the main objective of this study was to give an insight into the current measurement of treatment effectiveness in depression and to provide recommendations for its improvement.

**Materials and Methods:** The study was based on a multi-informant approach, comparing data from a systematic literature review, an expert survey with representatives from clinical practice (130), and qualitative interviews with patients (11) experiencing depression.

**Results:** Current literature places emphasis on symptomatic outcomes and neglects other domains of functioning, whereas clinicians and depressed patients highlight the importance of both. Interpersonal relationships, recreation and daily activities, communication, social participation, work difficulties were identified as being crucial for recovery. Personal factors, neglected by the literature, such as self-efficacy were introduced by experts and patients. Furthermore, clinicians and patients identified a number of differences regarding the areas improved by psychotherapeutic or pharmacological interventions that were not addressed by the pertinent literature.

**Conclusion:** Creation of a new cross-nationally applicable measure of psychosocial functioning, broader remission criteria, report of domain-specific information, and a personalized approach in treatment decision-making are the first crucial steps needed for the improvement of the measurement of treatment effectiveness in depression. A better measurement will facilitate the clinical decision making and answer the escalating burden of depression.

## Introduction

Clinical guidelines recommend antidepressant medication [selective serotonin reuptake inhibitors (SSRIs), serotonin–norepinephrine reuptake inhibitors (SNRIs) and tricyclic antidepressants (TCAs)] or psychotherapy (e.g., cognitive behavior therapy, interpersonal psychotherapy) as first choice treatment options for depression (McAllister-Williams, 2006; Patten et al., 2009). Results from randomized controlled trials and clinical guidelines suggest that internet based treatments and some complementary or alternative therapies, such as exercise or sleep deprivation, are also effective in the short term (Caliyurt and Guducu, 2005; Kvam et al., 2016). There is a large body of research on the effectiveness of these interventions in reducing depressive symptoms. Symptom improvement remains the main focus of clinical trials for depression, and the regulatory approval process for new medications and other interventions is based on symptomatology (Lam et al., 2015).

In spite of the large number of available interventions for depression and the huge evidence base on their effectiveness in terms of reducing symptom severity, the data show that more than 30% of all cases of depression are not adequately solved by first agent treatments (Kohn et al., 2004; National Collaborating Centre for Mental Health, 2010). The meta-analytical evidence of treatment effectiveness is also modest (Cuijpers et al., 2010; Khan and Brown, 2015). Moreover, depression has been ranked as one of the leading causes of burden in the Global Burden of Disease studies since 1990 (Whiteford et al., 2013). Some predictions indicate that it will be the greatest cause of disability worldwide by 2030 (World Health Organization, 2003). According to the World Health Organization (WHO), approximately 1 million people die from suicide every year (World Health Organization, 2003), and the majority of cases occur in the context of depression (Mann et al., 2005). In addition to the psychological burden on individuals, depression also has significant socio-economic costs. The direct and indirect costs of depression in the EU were estimated to be 92 billion in 2010 (Olesen et al., 2012). Nearly half of the costs were the result of productivity losses, indicating the enormous negative impact depression has on the economy.

Even though the lack of early detection and treatment of depression has been considered the main reason for the continuous burden of depression (World Health Organization, 2008), the lack of robust results poses the question of whether the current way of measuring depression is adequate or should be improved. Between 80 and 95% of all areas covered by the outcome measures in interventional studies represent clinical symptomatology (Brockow et al., 2004; McKnight and Kashdan, 2009; Kamenov et al., 2015). Other relevant areas of functioning beyond symptoms, such as activity limitations or participation restrictions in different domains of life, like social functioning and daily activities (World Health Organization, 2001), are mostly secondary outcomes and often do not account for systematic analyses (Williams et al., 2000). A number of studies state that these areas might more accurately predict the clinical course of depression (Stefos et al., 1996), whereas qualitative research shows that patients have prioritized these functioning outcomes over symptomatic outcomes and determined the return to a normal level of functioning at work, home or school as a major factor for remission in depression (Zimmerman et al., 2006a). Concurrently, some studies provide evidence that these outcomes do not correspond to symptom-based outcomes (Lam et al., 2015). It has been suggested that if symptoms provide early signs of treatment response, functioning outcomes beyond symptoms rather provide an indicator of meaningful change for the patient (McKnight and Kashdan, 2009).

Recently, the Canadian Network for Mood and Anxiety Treatments (CANMAT) highlighted the need for conceptualization and measurement of functioning outcomes in clinical trials (Lam et al., 2015). The lack of gold standard measures for assessing functioning has also been a major critique in recent studies (Lam et al., 2015; Madden et al., 2015). To fill this research gap, the WHO developed an evidence-based Core Set for depression (Cieza et al., 2004) to address the broad spectrum of functioning in depression. However, this tool has not been sufficiently implemented in research studies due to its complexity and large number of categories (Alvarezz, 2012). In addition, two instruments incorporating symptomatic outcomes, functioning, and quality of life (QoL) were created—the Individual Burden of Illness Index for depression (Cohen et al., 2013) and the Remission from Depression Questionnaire (Zimmerman et al., 2013)—but their validity is still insufficiently researched and therefore prevents broader usage in international research.

Thus, one of the potential reasons for the persisting burden of depression might be the lack of evidence on relevant and meaningful functioning difficulties for this disorder, possibly due to the lack of adequate functioning instruments (as mentioned above) or to the insufficient implementation of measures of functioning in clinical trials in general (Kamenov et al., 2015; Lam et al., 2015; Madden et al., 2015), which can assess comprehensively all areas affected by depression. This research gap was the impetus for the current study, which aimed to provide research recommendations for improving the measurement of treatment effectiveness in depression. More specifically, the study aimed to (1) provide information on the current areas included in the measurement of treatment effectiveness; (2) identify the areas that representatives from clinical practice and patients with depression consider relevant for inclusion in the assessment of psychotherapeutic, pharmacological or other complementary interventions; (3) compare the current status quo in research with the clinician and patient perspectives in order to identify the gaps in the measurement of treatment effectiveness; and (4) provide recommendations for its improvement and integration in future research.

To our knowledge, no previous study has focused on the improvement of treatment effectiveness measurement in depression. Such information would be very important for acquiring policy-relevant information on treatment effectiveness, disability, and rehabilitation, as well as for linking the available evidence to the best possible care of depressed patients.

## Materials and Methods

The present study was based on a comprehensive multi-informant approach, including data from a systematic literature review, expert clinicians in the field of depression, and patients currently diagnosed with depression. The opinion of clinical experts is essential for overcoming the gap between clinical research and the care of individual patients (Tonelli, 1999). However, qualitative research on patients living with a mental disorder was identified as one of the research priorities for public mental health in Europe (Forsman et al., 2015).

### Systematic Literature Review

Full details of the systematic literature review are provided elsewhere (Kamenov et al., 2015); a concise description is presented below. An electronic search for studies assessing interventions in depressive disorders was performed using four databases: PsycINFO, PubMed, Web of Science, and the Cochrane Central Register of Controlled Trials. Studies published between 2005 and 2015 were identified by including a set of sensitive MeSH terms and keywords indicative for intervention, depression and functioning. Studies were included if (1) participants were older than 18, (2) the diagnosis of depression was established by a standardized diagnostic tool, and (3) the sample included at least ten participants.

We grouped the treatments into three main categories: psychotherapy, pharmacotherapy, and “other” therapies, such as sleep deprivation and exercise therapy (Patten et al., 2009). All primary and secondary outcome measures assessing functioning, QoL or severity of symptoms that were already validated in depression samples were selected, and all individual items of the selected tools were extracted. The extracted items were analyzed and were linked to the International Classification of Functioning, Disability and Health (ICF) for operationalization purposes by applying the established linking rules (Cieza et al., 2005). The linking process was performed by two researchers. The items were grouped into 10 overarching categories based on the ICF classification. A frequency analysis was conducted after all functioning problems were identified to present the percentage of the areas stratified by type of intervention.

### Expert Survey

The survey was available between March 2015 and March 2016. It was designed to collect data from practicing clinicians in the field of depression, assessing interventions used in their daily practice and the psychosocial difficulties addressed by these treatments. The short survey consisted of two questions: (1) “Choose the type of intervention(s) you usually use in your daily practice”; and (2) “List the psychosocial difficulties that this intervention(s) aims to improve in individuals with depression.” Participants could choose up to ten interventions. The aim was to obtain experts’ opinion on the areas that are captured by the specific treatments and that should be included in the assessment of interventions for depression. All answers concerning psychosocial difficulties were linked to the ICF categories according to the existing rules (Cieza et al., 2005). Additionally, some demographic data were collected.

To reflect different opinions and achieve a maximum variation sampling, a wide range of clinicians were approached: psychiatrists, psychotherapists, primary health care doctors and other physicians, social workers, and nurses, amongst others. Clinicians were selected if they had at least 2 years of clinical expertise in depression. In addition, we searched for the highest possible variability in terms of age, gender, nationality, and type of therapy used in daily practice by the clinicians. Potential participants were identified through a number of sources—internal databases of international experts working in depression, heads of psychiatric hospitals and departments, professional websites for clinicians working in depression^1^ —and through a snowball approach. Experts were sent an email invitation for participation in the survey. We expected a 50–70% non-response rate (Archer, 2008; Horgan and Dimitriou, 2015). Our target was to obtain a sample of 100 experts, and therefore the survey was sent to nearly 250 experts in depression. The study aimed to approach European experts; therefore, the majority of clinicians resided in Europe. However, for comparison purposes, data from non-European experts were also collected.

### Qualitative Interviews with Patients with Depression

The aim of the present study was to collect data from individuals diagnosed with depression on the treatments they were receiving/had received for depression and the psychosocial difficulties addressed by the interventions. Therefore, qualitative individual interviews with outpatients diagnosed with depression were performed. Participants had to meet the following inclusion criteria: (1) current, or history of, depressive episode in the previous 12 months as main diagnosis (depressive episode [F32], recurrent depressive episode [F33], or currently in partial or total remission [F33.4] according to the International Classification of Diseases) (World Health Organization, 1992); (2) sufficient cognitive capacity to participate in an interview (score >26 on the mini-mental status examination (MMSE) (Folstein et al., 1975); (3) knowledge of the local language (Spanish); (4) age ≥18 years; (5) currently receiving treatment for depression, namely psychotherapy, pharmacotherapy or other treatment prescribed by their mental health professional; and (6) written informed consent. Ethics approval was obtained by the Hospital La Princesa Ethics Committee for Clinical Research in Madrid.

The recruitment of participants and data collection was performed in a public outpatient mental health unit at Hospital La Princesa (Madrid, Spain). Two mental health professionals working in the unit (one psychologist and one psychiatrist) collected the data between September 2015 and March 2016. All patients who met the inclusion criteria were informed about the study and invited to participate. All participants who agreed to participate gave their consent. The study consisted of a face-to-face interview with a research team member and comprised two parts. The first part was a series of demographic and clinical questions, which aimed to collect information on the patients’ background (e.g., gender, age, clinical information, such as diagnosis and severity of depression, number of previous episodes, onset of the disorder and occupation). The second part assessed the type of treatment (psychotherapeutic, pharmacological, or other) patients had received or were receiving, and their experience with the respective treatment. During an open personal interview, participants were asked about the psychosocial difficulties they were experiencing or had experienced in the past, and the ones that had or had not improved with the specific treatment they received. Data collection continued until a saturation point was reached. Saturation of data is a commonly used approach in qualitative research, indicating that there is a point in the analysis of data when sampling more data will not lead to more information related to the research questions (Glaser and Strauss, 1967). In this moment, researchers are allowed to stop sampling data and to round off the analysis. In the current study, the data collection continued until three consecutive patients in the same group of treatment (pharmacological or psychological) did not report new information, neither on the interventions received, nor on the psychosocial difficulties identified. When we reached the saturation point for both psychological and pharmacological interventions, we stopped with the recruitment of patients. Participants receiving psychotherapy were allowed to receive additional antidepressant pharmacotherapy when they met the following criteria: no antidepressant dosage change 1 month prior to the start of the psychotherapeutic sessions or during the psychotherapeutic treatment. As only two patients had been treated with other (alternative or complementary) therapies, no data on “other” therapies is available from this qualitative study.

Recordings of the individual interview sessions were transcribed verbatim. The transcripts were checked by the moderator and the information was extracted and double-checked. All content concerning psychosocial difficulties was translated into English and coded according to ICF categories following the existing ICF linking rules (Cieza et al., 2005). Codification of themes and subthemes for interventions and psychosocial difficulties was double-checked by an independent researcher and analyzed by NVIVO program, version 11. All frequencies were analyzed with SPSS, version 21.

## Results

### Study Characteristics

#### Literature Review

A total of 247 articles, including 71,904 participants, were included in the final synthesis. A total of 66 interventions were identified, all of them grouped into three main categories: psychotherapies (*N* = 22), pharmacotherapies (*N* = 20) or other therapies (*N* = 24). The most common intervention within the psychotherapeutic category was CBT. Fluoxetine in particular and the group of SSRIs in general were the most prevalent antidepressants. Among the remaining therapies, St. John’s wort was the first agent. A full summary of the study characteristics can be found elsewhere (Kamenov et al., 2015).

#### Expert Survey

The study was sent to 250 practicing clinicians, with a 52% response rate. 130 clinicians from around the world filled out the survey. 95 were professionals from 21 countries in Europe, and 35 (27%) were residing outside Europe. Among the non-Europeans, there were representatives from all continents, primarily from North and South America, with 15. The average age of the participants was 43 years (*SD* = 10.5, range: 23–65). Males were a slight majority (55%). Experts’ characteristics can be seen in **Table 1**.

**Table 1:** Characteristics of the experts (*N* = 130) participating in the online survey.

Variable	*N* (%)
**Age**	
18–34	32 (24.6%)
35–49	65 (50%)
50–64	31 (23.8%)
65+	2 (1.5%)
Females	59 (45%)
Years of experience mean (*SD*)	14 (10.23)
Non-European experts	35 (27%)
Psychiatrists	73 (56%)
Psychologists	44 (34%)
Others	13 (10%)


#### Qualitative Interviews with Patients

We conducted individual interviews with 11 patients who were receiving/had received in the last 12 months pharmacological or psychological treatment. Patients’ diagnoses varied from being in partial remission (*N* = 4) to experiencing a current mild (*N* = 2) or moderate episode (*N* = 5) of a major depressive disorder. The average age of the participants was 58 years (*SD* = 12), women were majority (*N* = 8). Patients’ characteristics can be seen in **Table 2**.

**Table 2:** Sociodemographic and clinical characteristics of the patients with depression.

Case	Age	Gender	Occupation	Diagnosis	Number of previous episodes	Age of diagnosis	Comorbidity
1	42	Female	Retired	Mild episode, currently in partial remission	0	40	Fibromyalgia
2	68	Female	Retired	Severe episode, currently in partial remission	1	64	No
3	48	Male	Currently working	Moderate episode, currently in partial remission	0	33	No
4	55	Female	Unemployed	Recurrent depression, currently in partial remission	10	25	Personality disorder
5	62	Female	Housewife	Recurrent depression, current moderate episode	2	55	No
6	86	Female	Retired	Recurrent depression, current moderate episode	No info	No info	No
7	54	Male	Currently working	Moderate episode	0	52	Psoriasis
8	60	Female	Currently working	Moderate episode	0	58	Psoriasis
9	65	Female	Housewife	Mild episode with somatic symptoms	1	53	No
10	48	Male	Unemployed	Moderate episode	2	38	HIV
11	55	Female	Unemployed	Mild episode	3	25	Cancer


### Comparison between Literature, Clinician, and Patient Perspectives on Treatment Effectiveness

Results from the literature review showed that items related to clinical symptoms—such as global mental functions (confidence, temperament, personality functions), specific mental functions (emotional functions, cognitive functions, body image), energy (energy level, appetite) and sleep functions—accounted for about 65% of the total number of areas addressed within the outcome measures. Body functions representing somatic symptoms (e.g., pain, digestive or sexual problems) accounted for an additional 15–18% across studies. Other areas of functioning beyond symptomatology; such as interpersonal relationships, leisure activities, daily tasks and demands; or major life areas, such as employment or education, represented a very small percentage: 15–20% varying across the categories of interventions. Domains such as social participation or communication represented a negligible percentage.

Unlike results from the literature review, expert clinicians gave minor importance to areas related to clinical symptoms (65%, varying across therapies – from 54% in psychotherapy to 67% in pharmacotherapy). To the contrary, the areas beyond symptomatology had higher importance compared to their role in the literature (from 30% in pharmacotherapy to 43% in psychotherapies). Interpersonal relationships, general tasks and demands, employment and education were pointed out by clinicians as fundamental areas. Communication and social participation represented a major part (up to 10%) of the functioning problems covered by therapies. There were no major differences between the areas identified and the types of therapy used by European and non-European experts.

**FIGURE 1:**
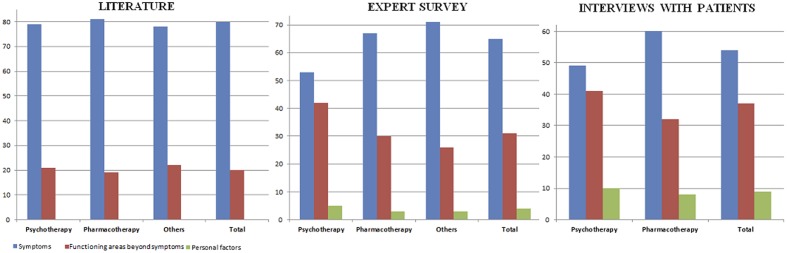
**Comparison between the percentages of functioning areas identified in the literature, the expert survey with clinicians, and individual interviews with patients with depression**.

The qualitative interviews with patients showed patterns similar to those of the expert reports. Patients highlighted the importance of a set of symptoms that represented 54% of all functioning areas. The importance of symptoms such as weight change or change in appetite was underlined by the participants: “*As something that has improved, I can point to my appetite. I have an appetite again and have gained weight. I lost five kilos and now I’ve regained them. I think the reason is that I feel more or less like I did before…*” (P8). However, the areas beyond symptomatology (37% of all areas identified) were also important for patients with depression. Interpersonal relationships were the only domain that was mentioned by all patients: “*Yes, now I go out and meet people. Before when I saw someone in a shop I would turn around and leave because I didn’t want him to stop me and talk to me*” (P2). Participants also highlighted problems at work, communication, and daily activities as crucial areas. A summary of all relevant areas found in literature, expert, and patient reports can be seen in **Figure 1**.

In addition, expert clinicians and patients identified a list of personal factors introducing the concept of “self”—self-perception, self-efficacy, self-acceptance, self-awareness, self-help, self-image and self-esteem—as a major part of the treatment process. These personal factors were neglected in the studies included in the systematic review, but constituted a relevant percentage in the answers of clinicians (4%) and patients (9%).

When the analysis was stratified by type of intervention, the literature did not reveal any differences among functioning areas included in studies applying psychotherapy, pharmacotherapy, or other interventions. However, such differences were found in the expert reports. Pharmacological treatment appeared to address symptomatic areas much more than psychotherapy (67% vs. 53%), whereas psychotherapy focused more on functioning areas beyond symptoms. Interpersonal relationships and communication constituted 30% of the total number of areas covered by psychotherapies, whereas both had a substantially smaller share in pharmacotherapy (7%) and other therapies (13%). Furthermore, communication represented 13% of the total number of areas covered by psychotherapy, but in pharmacological interventions it constituted only 2%. Likewise, in patient reports, symptomatic areas (54%) were predominantly reported by patients under pharmacological treatment. Lack of motivation for doing things was a salient area on the list of difficulties, with 80% of all patients reporting it as an area improved by medication. Regarding psychotherapeutic interventions, patients identified interpersonal relationships, communication, and emotional difficulties as the three main areas of improvement. More specifically, problems within the family, with an intimate partner or close friends were the most commonly reported improved difficulties: “*Also, I have my family around now. It affects me in a positive way, because it’s my family that I’m spending time with; I’m hiking in the mountain with my mother and my aunts. And this makes me happy*” (P4).

## Discussion

This study breaks new ground by identifying the drawbacks of the current measurement of treatment effectiveness in depression and by providing research recommendations for its improvement. This was done by comparing a systematic review of the literature, examining the areas of functioning included in the measurement process, and the clinician and patient perspectives on the actual areas addressed by the treatments. For comparison of the three sources of data, we adopted as a framework the ICF. The ICF provides a complete international standard language and coding system for data comparability. It is the most comprehensive classification for functioning including information about body functions and structures, activities and participation, as well as environmental factors that may have an impact on functioning. ICF provides a universal common language that allows us to operationalize the results and identify the differences between the research and the perspectives of patients and clinicians.

Our results showed that current research emphasizes symptomatic outcomes and neglects other domains of functioning, as opposed to the opinion of clinicians and depressed patients, who highlighted the importance of both. The “self” concept (e.g., self-efficacy, self-awareness), which was not considered in the literature, was introduced by experts and patients as a domain that can be improved by treatments and has a huge impact on the overall condition of individuals. Furthermore, clinicians and patients identified a number of differences regarding the areas improved by psychotherapeutic, pharmacological, and other treatments. Pharmacological treatments generally improved symptomatic domains to a higher extent. Experts expressed their preference in choosing antidepressants when targeting certain symptomatic difficulties, such as sleep or emotional functioning. Lack of motivation was an important issue for patients and they acknowledged the role of medications in its improvement. On the other hand, psychological treatments were the first choice for patients and experts when areas beyond symptoms were affected. Interpersonal relationships, problems in communication or lack of social participation were areas susceptible to change by psychotherapies. Self-care activities such as eating, dressing, taking care of one’s look were also recovered by psychological interventions. Based on the obtained results, the following section provides a summary of recommendations for the improvement of the measurement of treatment effectiveness in depression.

### Identification of the Most Burdensome Functioning Areas in Depression and Creation of a New Measure of Psychosocial Functioning

Our results show that a small percentage of studies apply instruments measuring functioning areas beyond symptoms. The few studies that implement such tools do not provide comprehensive information on functioning, mainly due to the limited number of areas covered by the instruments (Uebelacker et al., 2009; Vitriol et al., 2009). All clinicians and patients taking part in the present study identified a number of functioning domains (**Table 3**) as susceptible to change and crucial for assessment. This list of domains is the first step for the creation of a new instrument, which should include all relevant areas of psychosocial functioning, addressing together symptomatology and areas of functioning beyond symptoms. This unique tool should be comprehensive enough in regard to the health condition, and quick to administer if needed, to be applied in a clinical settings where practitioners have limited time and resources (Wittchen et al., 2001)— something that would provide comparability across studies. The instrument should also take into account differences across groups of patients by weighting the domains according to their importance. Thus, if for a certain group of patients who share similar characteristics, difficulties in communication and daily activities are prominent domains these should be given more weight than the other domains. However, our study is the first to assess this broad spectrum of psychosocial difficulties, and therefore more quantitative and qualitative research is needed to replicate our results and determine the most relevant domains of functioning.

**Table 3:** Functioning areas identified for inclusion in the measurement of treatment effectiveness.

Mental functioning	Global and specific mental functions. Represents symptoms such as emotional functions, rumination, anxiety, anhedonia, feelings of hopelessness and guilt, suicidal ideation, or impaired cognitive functioning
Sleep	Problems in the onset, maintenance and quality of sleep
Energy level	Fatigue, loss of energy and motivation
Somatic functioning	Somatic symptoms, pain or impaired sexual functioning
Interpersonal interactions and relationships	Relationships within the family, intimate relations, relations with friends, or informal social relationships
Recreational and leisure activities	Hobbies, socializing, sports, arts and culture
Communication and social participation	Problems in communication, receiving and producing messages, participation in society, social activities, etc.
General tasks and demands	Problems in daily activities, household responsibilities, self-care, handling stress
Major life areas	Employment, education, economic life
Personal factors	Self-esteem, self-efficacy, self-image, self-awareness


Another approach that can facilitate functioning data collection is the application of patient-generated outcome measures or the integration of patient-generated individual items in a patient-reported tool. The patient-generated information is a novel approach to evaluating outcomes that allows patients to formulate their own responses in an open-ended format based on each patient’s own stated goals and expectations (Tang et al., 2014). Even though this approach has certain limitations: e.g., it requires more extensive resources for developing and training personnel to score answers in a standardized format, and is less amenable for deriving comparisons across populations, it can provide valuable information on the living experience of depression and therefore should be given the necessary attention.

### A Cross-Nationally Applicable Measure of Functioning

The majority of studies included in the systematic review provided evidence only from high-income countries. Knowledge on relevant areas in depression from low- and middle-income countries is sparse. The results from the expert survey did not reveal major differences in the answers of European and non-European clinicians; however, these results are not generalizable due to the small number of non-European clinicians. This lack of evidence suggests that a new instrument comprehensively assessing all relevant functioning areas should be also validated in different cross-national samples. Moreover, the instrument should be sensitive to country differences and be validated in different settings. More research from low-, middle-, and high-income countries is needed to provide country-specific functioning information.

### Broader Remission Criteria

Remission of depression is currently defined solely in terms of symptom reduction (Zimmerman et al., 2006b) according to cut-off scores on symptom severity scales, such as the Hamilton Rating Scale for Depression (HRSD) (Hamilton, 1967), Beck Depression Inventory (BDI) (Beck et al., 1961) or the Montgomery–Åsberg Depression Rating Scale (Montgomery and Asberg, 1979). A more comprehensive definition of remission is needed to adequately reflect the experience of depressed patients under treatment. Our results show that improvement in functioning areas beyond symptoms is as important as the reduction in symptomatology. One possibility is the creation of a new instrument covering not just symptomatic aspects, but all relevant affected areas. There are already initial steps in this direction. Cohen et al. (2013) created an Individual Burden of Illness Index for depression to measure treatment impact and recovery in depression by incorporating multidimensional patient-reported outcomes of symptom severity, functioning, and QoL. Zimmerman et al. (2014) subsequently validated a new instrument: the Remission from Depression Questionnaire, encompassing different domains of functioning and QoL, along with symptomatology. These authors conclude that their new tool provides a broader perspective on depressed patients’ condition than purely symptom-based measures and is more consistent with the biopsychosocial approach in the treatment of depression. However, these tools are still in their infancy and need further validation. Another possibility involves a separate definition of functional remission alongside symptom assessment. An example is a study by Mancini et al. (2012), which applied such criteria, based on the Sheehan Disability Scale (Sheehan, 1983). Future studies should aim to achieve such broader remission criteria.

### Reporting Domain-Specific Information Rather than Sum-Scores of Questionnaires for Functioning

Results from the literature review showed that more than 80% of the interventional studies published in the last decade reported only sum-scores of instruments assessing functioning rather than domain-specific information. Despite some methodological and practical advantages of aggregating scores from different domains, these sum-scores also obscure potential differences among people and do not provide detailed information on the differential impact of certain functioning domains on the overall state of depressed persons. A higher sum-score might mean a higher number of less affected functioning areas or a smaller number of domains with marked deterioration. Reporting domain-specific information will potentially reveal differential trajectories in the course of depression, interrelations between distinct domains of functioning, and most importantly, will lead to a more personalized approach in the treatment of depression.

### Personalized Approach in Treatment Decision Making

Current treatment decision making is primarily based on evidence-based medicine. Thus, clinical guidelines recommend psychotherapy and pharmacological agents for all patients as first-line treatments. The regulatory approval process for new medications and other interventions is based primarily on symptomatology. Our results, however, showed that psychotherapeutic and pharmacological interventions targeted the range of functioning difficulties in the population to a different extent. Moreover, patients and experts highlighted the importance of functioning difficulties beyond symptoms in the recovery process. There is a need for a more personalized approach in treatment decision-making that acknowledges specific patient needs and accounts for a more comprehensive array of functioning domains. More research is also needed to explore the effectiveness of the available interventions in each of the relevant functioning areas.

Even though the present study considered all possible perspectives on the measurement of treatment effectiveness, some methodological limitations should be mentioned. First of all, the literature review covered only the last 10 years of research, because we aimed to explore the latest trends in assessing treatment effectiveness. Secondly, approximately 70% of the clinicians that took part in the online survey were European. Even though we achieved a representation of non-European experts, wider participation of the latter may have provided different perspectives on the topic. Finally, data was obtained from only 11 patients from Spain. Our approach was the attainment of a saturation point in individuals’ answers, but more patients, with different cultural backgrounds, could have enriched the data. The qualitative study was the first to our knowledge to explore such wide array of functioning difficulties addressed by different interventions. Furthermore, the use of the ICF classification system as a framework allowed the comparison between patients’, practitioners’ and literature perspectives. Therefore, we think that despite the small number of participants in the qualitative study, our findings are promising and warrant further investigation.

The present study is the first to our knowledge to provide recommendations for improved treatment measurement using a methodology based on a multi-informant approach. Clinician and patient perspectives are essential for informing the context of clinical research, and overcoming the gap between clinical research and the care of individual patients. We believe that more accurate and comprehensive evidence on the effectiveness of available interventions for depression is needed to answer the steadily escalating societal and economic burden of the disease.

## Author Contributions

KK, MC, MN, RB, EK, CR-K, and JA-M conceived, designed, and performed the experiments. KK and MC analyzed the data. KK wrote the paper. MC, MN, RB, EK, CR-K, and JA-M made critical revisions of the manuscript for important intellectual content.

## Conflict of Interest Statement

The authors declare that the research was conducted in the absence of any commercial or financial relationships that could be construed as a potential conflict of interest.

## Footnotes

1 http://www.commonlanguagepsychotherapy.org
